# Interaction of Aβ42 with Membranes Triggers the Self-Assembly into Oligomers

**DOI:** 10.3390/ijms21031129

**Published:** 2020-02-08

**Authors:** Siddhartha Banerjee, Mohtadin Hashemi, Karen Zagorski, Yuri L. Lyubchenko

**Affiliations:** Department of Pharmaceutical Sciences, University of Nebraska Medical Center, 986025 Nebraska Medical Center, Omaha, NE 68198-6025, USA; siddhartha.banerjee@unmc.edu (S.B.); mohtadin.hashemi@unmc.edu (M.H.); karen.zagorski@unmc.edu (K.Z.)

**Keywords:** amyloid aggregation, supported lipid bilayers, time-lapse AFM, Alzheimer’s disease, molecular dynamics simulation

## Abstract

The self-assembly of amyloid β (Aβ) proteins into oligomers is the major pathogenic event leading to Alzheimer’s disease (AD). Typical in vitro experiments require high protein concentrations, whereas the physiological concentration of Aβ is in the picomolar to low nanomolar range. This complicates the translation of results obtained in vitro to understanding the aggregation process in vivo. Here, we demonstrate that Aβ42 self-assembles into aggregates on membrane bilayers at low nanomolar concentrations - a pathway in which the membrane plays the role of a catalyst. Additionally, physiological ionic conditions (150 mM NaCl) significantly enhance on-membrane aggregation, leading to the rapid formation of oligomers. The self-assembly process is reversible, so assembled aggregates can dissociate from the membrane surface into the bulk solution to further participate in the aggregation process. Molecular dynamics simulations demonstrate that the transient membrane-Aβ interaction dramatically changes the protein conformation, facilitating the assembly of dimers. The results indicate peptide–membrane interaction is the critical step towards oligomer formation at physiologically low protein concentrations.

## 1. Introduction

Growing evidence suggests the involvement of protein oligomers in the development of protein misfolding diseases, including Alzheimer’s disease (AD) and Parkinson’s disease (PD); however, limited knowledge exists regarding the molecular mechanisms behind the aggregation processes for these oligomers [1,2,3,4]. The amyloid cascade hypothesis (ACH), proposed more than a quarter-century ago [5], is the major model used to describe the pathology of AD and other neurodegenerative diseases [5,6,7,8,9]. ACH posits that the onset of diseases involves the spontaneous assembly of an amyloidogenic polypeptide. In turn, the accumulation of aggregates defines the disease state. Translational studies in the framework of ACH are focused on decreasing the concentration of amyloid proteins to decelerate the aggregation process (reviewed in [6,7,10]). However, drug development efforts based on decreasing the amyloid β (Aβ) concentration, as well as disaggregating the plaques, have failed [11,12], which challenges the validity of ACH [13]. In fact, there are a number of problems with ACH [7], one being the concentration of Aβ. Specifically, in vitro aggregation experiments require Aβ concentrations in the micromolar range, whereas Aβ levels in brain and cerebral spinal fluid (CSF) are frequently in the low nanomolar range [10,14,15,16]; this value remains in the same range regardless of the disease state [17,18]. Therefore, an explanation is needed as to how Aβ proteins at such low concentrations can assemble into aggregates. It is also unknown what causes the amyloid protein to aggregate. These problems with ACH have driven alternative models for the development of AD [10].

We have recently discovered that amyloid proteins, including amyloid β 42 (Aβ42) and its Aβ (14–23) segment, are capable of assembling into aggregates in the nanomolar concentration range at the surface–liquid interface [19,20,21]. We developed a model, according to which monomers that were transiently immobilized on surfaces increased the local protein monomer concentration, and thus worked as nuclei to dramatically accelerate the entire aggregation process [21]. This theoretical model was verified by experimental AFM studies, and we termed this aggregation pathway the surface-catalyzed aggregation process. We have recently examined whether the surface catalysis process can be extended to membrane surfaces. We selected α-synuclein (α-syn) and performed time-lapse AFM studies [20,22]. We used supported lipid bilayers (SLBs) formed by 1-palmitoyl-2-oleoyl-glycero-3-phosphocholine (POPC) and 1-palmitoyl-2-oleoyl-sn-glycero-3-phospho-l-serine (POPS), which are major components of neuronal membranes. We demonstrated that SLBs catalyze the aggregation of α-syn at concentrations as low as 10 nM, which corresponds to the concentration range in the CSF [23]. The aggregation kinetics were also found to be dependent on the SLB composition, being considerably greater for the POPS bilayer compared to POPC. Computational modeling suggested that α-syn monomers changed conformation upon interaction with the bilayers, and these interactions were dependent on the composition of the bilayer. The conformations of α-syn after binding to POPS dramatically facilitated assembly into the dimer, a property that is in contrast with POPC and in line with experimental data. Note that a similar phenomenon has been reported for another amyloid protein—transthyretin—which causes systemic amyloidosis and leads to a variety of tissue damage [24,25].

In the present study, we applied the same combined experimental and computational approaches to characterize the aggregation of Aβ42 at its physiologically relevant low concentration (10 nM). The time-lapse AFM imaging revealed Aβ42 aggregation on the bilayer surfaces, while no self-assembly of Aβ42 was detected in bulk solution. The assembled aggregates were not strongly bound to the surface and were capable of spontaneous dissociation into solution. Importantly, the self-assembly process did not cause damage to the surface, as no defects were detected after the aggregates were dissociated. We performed computational modeling of the aggregation process on the membrane surfaces and demonstrated that interaction with the membrane dramatically changes the conformation of Aβ42 monomers. Moreover, membrane-bound Aβ42 proteins trigger the assembly of dimers, propagating the misfolded states of the Aβ42 molecules. Thus, interaction with membranes results in the transition of Aβ42 into the aggregation-prone, misfolded conformations. Such conformations have not been reported in simulations or experiments performed in bulk solutions. Given that the membrane-assembled aggregates can dissociate into solution, the recently discovered on-membrane aggregation can be the mechanism by which amyloid oligomers, or their disease-prone seeds, are produced and spread over the organism.

## 2. Results

### 2.1. Experimental Studies

#### 2.1.1. Aggregation on Supported Lipid Bilayers (SLB)

We used time-lapse AFM to directly visualize the aggregation process of the Aβ42 protein on SLBs formed from POPC and POPS. The key in these studies was the assembly of a topographically smooth bilayer that remains stable over the entire observation period, which typically was over several hours. Freshly cleaved mica was used as the support substrate. We developed a method which is described in papers [19,20,22,26]. Typical examples of AFM images of POPC, POPS and POPC:POPS bilayers are shown in Figure S1. The surface was smooth over several microns, with a few defects that did not change over time. These defects were used to measure the thickness of the bilayer. Cross-section profiles in this figure reveal the height value of 4.2–4.5 nm, validating the assembly of the bilayer. The smooth surface topography remains stable during hours of observation, regardless of the bilayer composition.

#### 2.1.2. Aggregation on the POPC Bilayer

**Figure 1:**
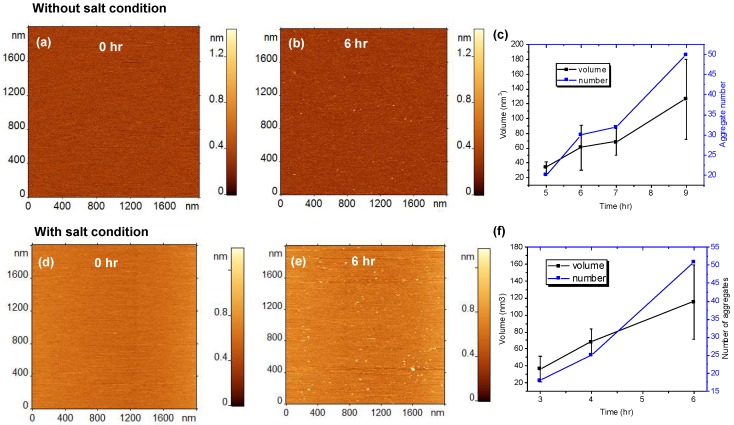
Aggregation of 10 nM amyloid β 42 (Aβ42) on 1-palmitoyl-2-oleoyl-sn-glycero-3-phosphocholine (POPC) supported lipid bilayer (SLB). (**a**–**c**) Show the results obtained in the absence of 150 mM NaCl in the buffer (without salt condition) and (**d**–**f**) show the aggregation results with 150 mM NaCl (with salt condition). (**a**) AFM topographic image of POPC SLB just after the addition of Aβ42 onto the SLB surface. (**b**) The same SLB surface after incubating for 6 h, without 150 mM NaCl. The small globular features indicate the presence of aggregates. (**c**) The plot shows the increase in number and volume of the aggregates with time. (**d**–**f**) show similar experiments but with 150 mM NaCl added to the buffer.

After the assembly of the bilayer and its inspection with AFM, a solution of 10 nM Aβ42 in 10 mM sodium phosphate (pH 7.4) was placed on top of the SLBs and imaged at room temperature. Figure 1a shows the surface topography taken just after the injection of protein sample. The surface remains smooth; however, we cannot exclude the binding of Aβ42 monomers, as these are too small (~4 kDa) for reliable visualization with AFM [19]. The time-dependent aggregation of 10 nM Aβ42 on the POPC bilayer is shown in Figure 1a–c, with Aβ42 aggregates appearing on the SLB after 6 h. The time-lapse experiment was continued up to 9 h. The quantitative analysis of the number and sizes of aggregates, shown in Figure 1c and Figure S2a, demonstrate that these parameters increase gradually over time, whereas no aggregation in the bulk solution was observed, even after 48 h [19].

We also tested how ionic conditions close to physiological ones affect the aggregation propensity on the POPC bilayer by performing time-lapse AFM experiments in the presence of 150 mM NaCl. The results are shown in Figure 1d–f. Frame d shows the clean surface just after the addition of Aβ42, and frame e shows a substantial amount of aggregates after 6 h of incubation. Figure 1f shows the gradual increase in both the number and volume of the aggregates in presence of NaCl. Quantitative analysis (Figure 1c,f and Figure S2a,b) demonstrates that aggregates with volumes ~120 nm^3^ appear after 6 h in the presence of 150 mM NaCl, while in the absence of NaCl similar aggregates are formed after 9 h. These data suggest that physiological ionic conditions further increase the aggregation propensity on the POPC bilayer.

#### 2.1.3. Aggregation on the POPS Bilayer

**Figure 2:**
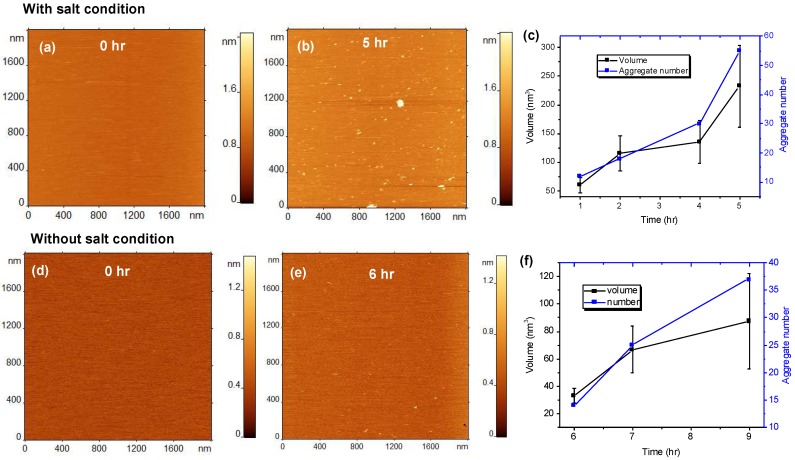
Aggregation of 10 nM amyloid β 42 (Aβ42) on 1-palmitoyl-2-oleoyl-sn-glycero-3-phospho-L-serine (POPS) supported lipid bilayer (SLB). (**a**–**c**) The results obtained in the presence of 150 mM NaCl in the buffer (with salt condition) and (**d**–**f**) shows the aggregation results without 150 mM NaCl (without salt condition). (**a**) AFM topographic image of POPS SLB just after the addition of 10 nM Aβ42 onto the SLB surface. (**b**) The same SLB surface having Aβ42 aggregates after incubating for 5 h with 150 mM NaCl (with salt condition). The small globular features indicate the presence of aggregates. (**c**) The plot shows the increase in number and volume of the aggregates with increase in time. (**d**–**f**) show similar aggregation experiments but without 150 mM NaCl in the buffer.

Next, we tested how critical the composition of the bilayer is for the on-surface catalysis of aggregation of Aβ42. We performed time-lapse aggregation studies for the negatively charged POPS SLB in the presence of 150 mM NaCl. The results are shown in Figure 2a–c. Frames a-b represent typical time-lapse images of the aggregates assembled by 10 nM Aβ42 on the POPS SLB. After 5 h of time-lapse imaging, a significant amount of aggregates are observed on the SLB surface (Figure 2b). The time-dependent values of the number and volumes of aggregates are plotted in Figure 2c and Figure S2d; they demonstrate that both values increase gradually with time. A comparison of the data obtained for POPC and POPS bilayers (Figure 1e and Figure 2b) indicates that the aggregates are large on the negatively charged POPS surface compared to those on POPC. To identify the effect of NaCl on the aggregation kinetics of Aβ42 on POPS SLB, we then performed a similar experiment but with salt excluded from the buffer. Similar to POPC, the aggregation process is slower without 150 mM NaCl (Figure 2e and Figure S2c). Figure 2e shows an AFM image of the POPS surface after incubation of 10 nM Aβ42 without salt. The surface contains a substantially smaller number of aggregates compared to the salt condition (Figure 2b). Figure 2f shows the increase in number and volume of aggregates with time.

#### 2.1.4. Aggregation on Binary SLB Mixture (POPC:POPS)

**Figure 3:**
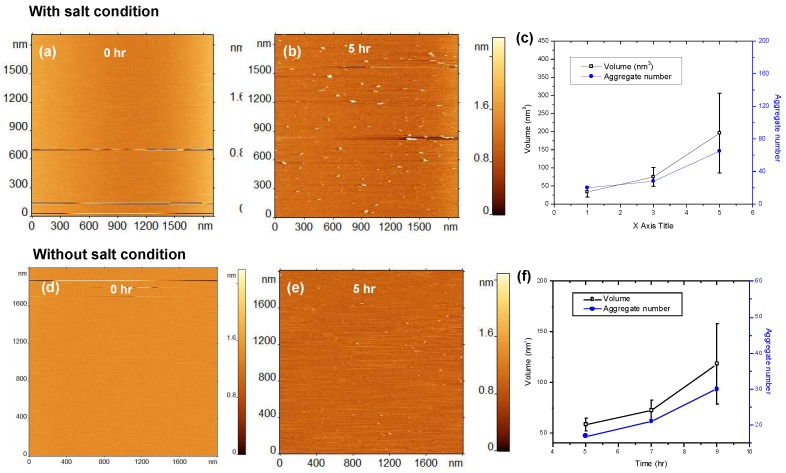
Aggregation of 10 nM amyloid β 42 (Aβ42) on 1-palmitoyl-2-oleoyl-sn-glycero-3-phosphocholine:1-palmitoyl-2-oleoyl-sn-glycero-3-phospho-L-serine (POPC-POPS) supported lipid bilayer (SLB). (**a**–**c**) Show the results obtained in the presence of 150 mM NaCl in the buffer (with salt condition) and (**d**–**f**) show the aggregation results without 150 mM NaCl (without salt condition). (**a**) AFM topographic image of POPC-POPS SLB just after the addition of Aβ42 onto the SLB surface. (**b**) The same SLB surface having Aβ42 aggregates after incubating for 5 h with 150 mM NaCl in the buffer (with salt condition). The small globular features indicate the presence of aggregates. (**c**) The plot shows the increase in number and volume of the aggregates with increase in time. (**d**–**f**) show results from similar experiment but without 150 mM NaCl in the buffer.

To further characterize the effect of the bilayer composition on the aggregation catalysis, similar time-lapse AFM experiments were performed for the SLB formed by an equimolar mixture of POPC and POPS in presence of 150 mM NaCl. The results are shown in Figure 3a–c. As observed in the previous experiments, aggregates appeared on the surface, and after 5 h the surface contained a significantly higher number of aggregates (Figure 3b) compared to the same surface without salt present (Figure 3e). The sizes of the aggregates in the presence of NaCl, are also larger compared to the no salt condition. The aggregate size after 5 h of incubation without salt was ~60 nm^3^ (Figure 3f), whereas similar sized aggregates were observed within 1 h of incubation in the presence of NaCl (Figure 3c). These results clearly indicate that the presence of NaCl strongly influences the acceleration of aggregation and becomes an important factor allowing aggregates to grow faster.

**Figure 4:**
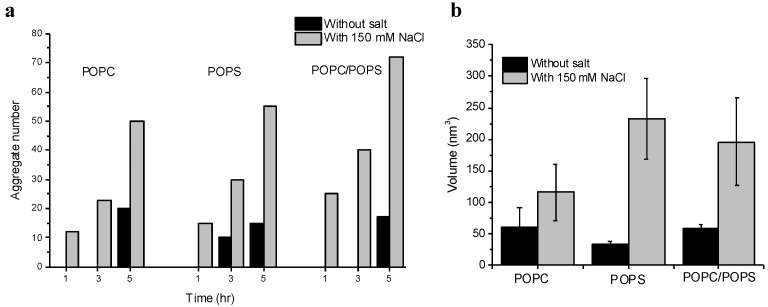
Volume and number of aggregates formed on different supported lipid bilayer (SLB) surfaces. (**a**) Comparison of number of aggregates formed by 10 nM Aβ42 after 1 h, 3 h, and 5 h of incubation with 150 mM NaCl (gray bars) and without NaCl (black bars) on the three SLB surfaces. The missing black bars at different time-points indicate no aggregates were visible under those conditions. (**b**) Comparison of the volume of aggregates formed on 1-palmitoyl-2-oleoyl-sn-glycero-3-phosphocholine (POPC), 1-palmitoyl-2-oleoyl-sn-glycero-3-phospho-l-serine (POPS), and POPC-POPS SLBs with and without salt condition after 5 h of incubation. The volume of the aggregates formed after 5 h of incubation is much larger in presence of NaCl on all three surfaces.

A quantitative comparison of aggregation on the three bilayer surfaces has been done and the data is assembled as histograms, shown in Figure 4. The cumulative plot in Figure 4a shows the number of aggregates observed on the three different SLB surfaces after 1, 3, and 5 h of incubation with 10 nM Aβ42. The grey bars represent the condition with 150 mM NaCl in the buffer and black bars shows the no salt condition. The missing black bars for some time-points indicate that no substantial number of aggregates were observed at that time-point. For example, no aggregates were visible after 1 h and 3 h on POPC without salt (missing black bars), but aggregates were present on the POPC surface (gray bars) with NaCl at those time-points. Overall, aggregates were visible on all three surfaces after 1 h in presence of NaCl, but the aggregates appeared later without the salt condition. Figure 4b summarizes and compares the size of the aggregates formed on the three SLB surfaces after 5 h of incubation with and without salt. In all three cases, the presence of NaCl promotes the formation of larger aggregates, indicating the role of NaCl in the on-membrane aggregation of Aβ42.

#### 2.1.5. Dissociation of Aggregates from the Bilayer Surface

**Figure 5:**
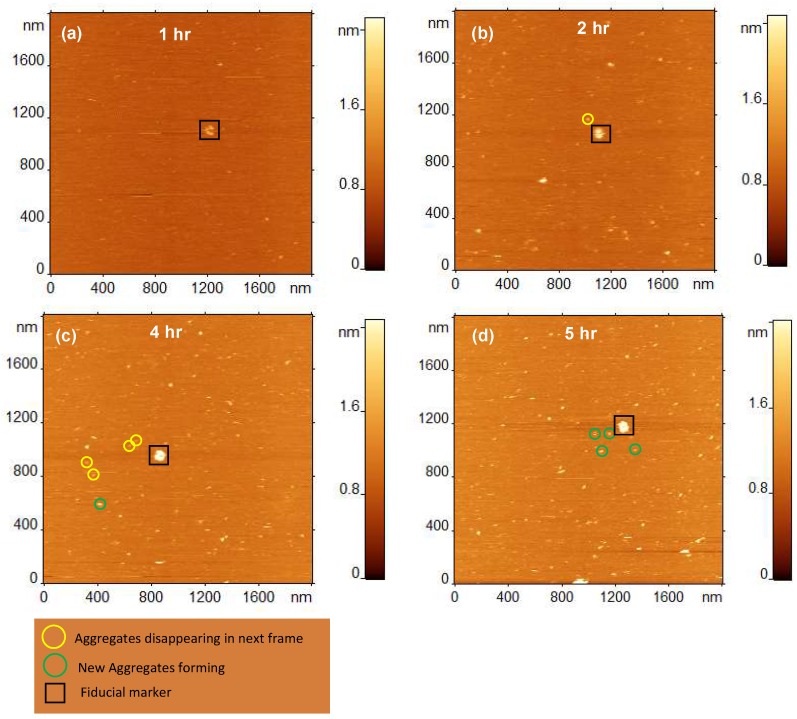
Dynamics of aggregate formation and dissociation from 1-palmitoyl-2-oleoyl-sn-glycero-3-phospho-l-serine (POPS) supported lipid bilayer (SLB) surface. (**a**) Time-lapse AFM imaging of 10 nM amyloid β 42 (Aβ42) on POPS SLB after 1 h incubation. (**b**–**d**) Same area of the surface scanned at the noted time-points. Black square depicts an aggregate that is present in all frames and acts as a fiducial marker. Aggregates circled in yellow are desorbed from the surface between frames, whereas those circled in green are newly formed on the surface.

Time-lapse AFM experiments allowed us to directly monitor the assembly-disassembly process for each aggregate (Figure 5a–d). The data show that aggregates formed on the surface are able to dissociate from the surface. The black square in frames “a–d” indicates a feature that is present in all frames and acts as a fiducial marker. This allows us to detect the relative position of newly formed aggregates and desorption of already formed aggregates. The aggregates marked with yellow circles are absent in the next frame, while aggregates marked with green circles have appeared. For example, the aggregate circled in yellow in frame “b” is gone in frame “c”, whereas, the aggregate circled in green in frame “c” has appeared and was absent in frame “b”. These images demonstrate that assembled Aβ aggregates can dissociate from the surface and go into the solution.

In case the dissociation of aggregates can lead to their accumulation in the solution, the concentration of aggregates above the bilayer should grow. To test this theory, aliquots from the solution above the SLB were taken, deposited on mica, imaged, and analyzed. A set of images corresponding to aliquots from different time-points demonstrates the accumulation of aggregates in the solution above the SLB, shown in Figure S3. Frames shown in Figure S3a depict representative images of aliquots taken from the solution above the SLB surface, whereas frames in Figure S3b depict the control experiment without SLB surface. The plot (Figure S3c) shows a substantial amount of aggregate accumulation in 6 h and 24 h in the solution above the SLB, whereas only a few aggregates appear in the control.

### 2.2. Computer Simulations of Aβ42 Interacting with Bilayers

To determine the underlying mechanism of interaction between Aβ42 and lipid bilayers, we employed extensive microseconds-long molecular dynamics (MD) simulations. Briefly, POPC and POPS bilayers were assembled using 512 lipids, solvated with 40:1 water:lipid, and neutralized using NaCl. After energy minimization, each bilayer system was then simulated for 150 ns to obtain a relaxed bilayer. Aβ42 monomer or dimer, obtained from [27], were then added to the relaxed bilayers at a distance of 5 nm from the bilayer centers. The systems were then neutralized and kept at 150 mM ion concentration using NaCl counterions. Following short preparatory simulations, each system was then run on the special purpose Anton 2 supercomputer for 5 μs each and the interaction of the Aβ molecules with the bilayers was recorded. All simulation system configurations and durations are presented in Table 1.

#### 2.2.1. Interactions of Aβ42 Monomer with POPC

**Figure 6:**
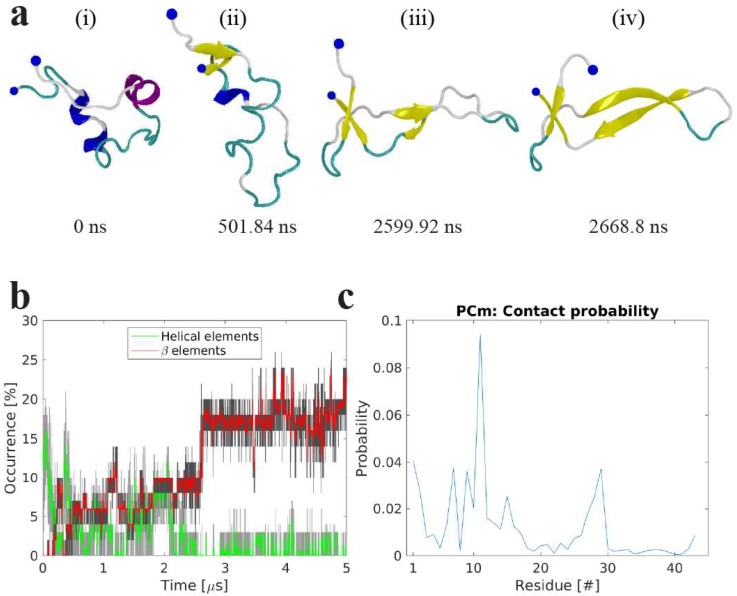
Molecular dynamics simulations of the interaction of amyloid β 42 (Aβ42) monomer with 1-palmitoyl-2-oleoyl-sn-glycero-3-phosphocholine (POPC) bilayer. (**a**) Snapshots showing the change of Aβ42 conformation and secondary structure at different time-points while interacting with the POPC bilayer. Protein is depicted as a cartoon following VMD coloring scheme (yellow β-strands and purple α-helices), N- and C-terminal Cα are presented as a large and a small blue sphere, respectively. (**b**) The evolution of secondary β-structure (β-sheet and β-bridge, red), and helical (α-, π-, and 3/10-helices, green), elements of the Aβ42 protein as determined by DSSP. The graphs are moving averages using a 1 ns window; raw data is presented as dark and light grey graphs, respectively. (**c**) Per residue contact probability plot between residues of the Aβ42 monomer and the P atoms of the POPC headgroups.

Upon and during interaction with the POPC bilayer, the Aβ42 monomer experiences structural and conformational changes. Figure 6a shows snapshots illustrating the transition of the Aβ monomer induced by interaction with the POPC bilayer. The initial conformation of the Aβ monomer (snapshot (i)), taken from our recent paper [27], is a compact collapsed conformation with two small helical regions, shown as blue and purple ribbons, and an otherwise essentially random coil. The conformation gradually transitions to an elongated shape after ~ 500 ns, and two β-strands (yellow arrows) appear at the C- and N-termini of the molecule (snapshot (ii)). This elongated conformation remains stable for approximately 2 μs; however, the β-strand segments increase in size. This is then followed by the formation of two β-strands in the central region of the molecule (snapshot (iii)). A cooperative transition within the monomer then occurs, accompanied by the formation of a β-hairpin structure (snapshot (iv)); this structure remains stable until the end of the 5 μs-long simulation. The structural transition of the Aβ monomer induced by the interaction with the POPC bilayer is shown graphically in Figure 6b and can be viewed in Movie S1. The monomer starts with negligible β-structure content and ~12% helical content, but rapidly transitions to conformations with 5–10% β-content (after approximately 500 ns), followed by an abrupt transition at around ~2.5 μs to ~17% β-content that remains for the remainder of the simulation. Importantly, such dramatic conformational transitions within the Aβ monomer are induced by transient interactions between the POPC surface and peptide residues, with the primary interactions being through residues 7–12 and 27–29; these segments also experience the largest change in structure and adopt β-strands during the simulation (Figure 6c).

#### 2.2.2. Interactions of Preformed Aβ42 Dimer with POPC

**Figure 7:**
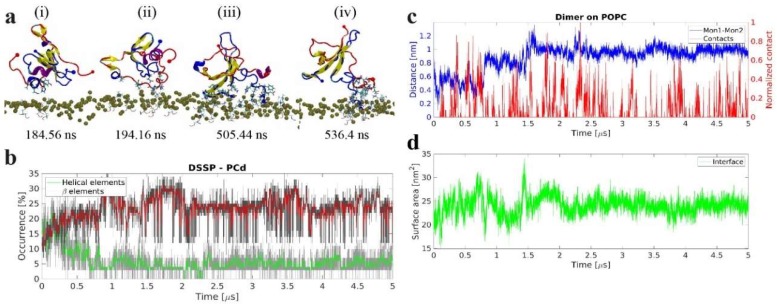
The dynamics of interactions between preformed dimer and 1-palmitoyl-2-oleoyl-sn-glycero-3-phosphocholine (POPC) bilayer. (**a**) Snapshots showing conformational change of preformed amyloid β 42 (Aβ42) dimer upon interaction with the POPC bilayer. Protein side-chains and POPC head groups are shown for interacting residues. Protein is depicted as cartoon following VMD coloring scheme, N- and C-terminal Cα are represented as large and small spheres in blue and red for Mon 1 and Mon 2, respectively, POPC P atoms are depicted as gold spheres. (**b**) Evolution of β-structure (β-sheet and β-bridge, red), and helical (α-, π-, and 3/10, green), elements of the preformed dimer over time. The graphs are moving averages using a 1 ns window; raw data is presented as dark and light grey graphs, respectively. (**c**) Center of mass (CoM) distance between the two monomers within the preformed dimer, blue, and normalized contacts between the dimer and the lipid headgroups, red. (**d**) Time evolution of the dimer interface surface area depicting the total area of interaction between the monomers.

We then investigated whether interactions with the POPC bilayer have an effect on the structure of Aβ42 dimer assembled in bulk solution, and if these interactions are capable of changing the conformation of the dimer. Aβ42 dimer, with the structure identified in [27], was placed above the POPC bilayer and the results of MD simulations are summarized in Figure 7. Snapshots in Figure 7a demonstrate a rapid formation of β-sheet within the central and C-terminal segments of one monomer (i), followed by the β-strand formation in C-terminal segment in the other monomer (ii). This strand is further extended and becomes a nine-residue strand that remains stable until the end of the simulation (iii–iv). Graphically, this is depicted in Figure 7b, which shows the evolution of secondary structure elements versus time. Initially the dimer has <10% β-structure content, which over time gradually increases to ~20%; on the other hand, the helical content decreases with time.

The preformed Aβ42 dimer remains stable during the entire simulation, but experiences conformational change during and following interactions with the bilayer, as seen on Figure 7c. This is clearly observed from the change in center of mass (CoM) distance between the two monomers within the dimer, seen in Figure 7c. The figure also shows that the greatest change occurs when the dimer is in contact with or immediately after being in contact with the bilayer. The reorganization of the dimer also changes the interfacial area between the two monomers; initially ~20 nm^2^, it increases to approximately 25 nm^2^, shown in Figure 7d. Furthermore, as the dimer interacts with the bilayer, the residues forming the dimer interface also change, seen in Figure S4a. Initially, the dimer is stabilized by contacts between the N-termini and the central regions (i); however, as the dimer conformation changes, the interactions shift to also include residues of the C-termini (ii) before finally shifting to interactions between the C–C and C–N termini (iii). The probability of a residue to interact with the POPC membrane is very similar for the two monomers within the dimer, with the N-terminal and residues 29–31 having the highest probability to interact with the membrane, shown in Figure S4b.

#### 2.2.3. Dimerization on POPC Bilayer

**Figure 8:**
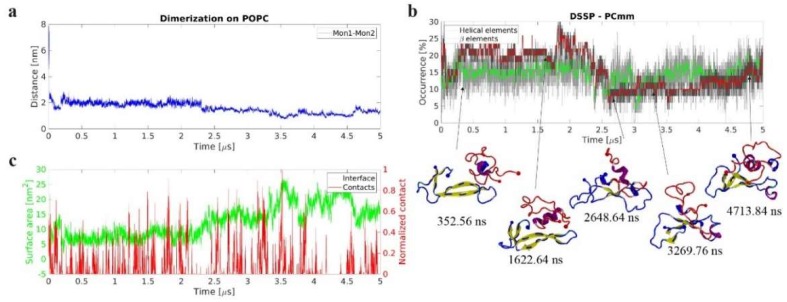
Dynamics of on-surface dimerization of amyloid β42 (Aβ42) monomers in presence of 1-palmitoyl-2-oleoyl-sn-glycero-3-phosphocholine (POPC) bilayer. (**a**) Time-dependent center of mass (CoM) distance between the two Aβ42 monomers revealing that the dimer rapidly forms and remains stable. (**b**) Evolution of secondary structure β-structure (β-sheet and β-bridge, red), and helical (α-, π-, and 3/10, green), elements, as determined by DSSP. The graphs are moving averages using a 1 ns window; raw data is presented as dark and light grey graphs, respectively. Key transition points are highlighted with cartoon representation of the protein structures. Initial surface-bound monomer is depicted in blue and the initially free Aβ42 monomer in red. N- and C-terminal Cα are large and small spheres, respectively. Secondary structure elements are depicted using VMD color scheme (yellow β-strands and purple α-helices). (**c**) Dimer interface surface area, green, and normalized number of contacts between Aβ42 and lipid headgroups, red, showing evolution and correlation between these parameters.

To elucidate the role of conformational transitions within the Aβ monomer in the aggregation process, we modeled the assembly of a dimer by the interaction of an Aβ monomer at the surface with a free monomer. Briefly, we selected the last frame of the monomer-POPC simulation (Figure 6) and added another monomer at 4 nm CoM distance with respect to the membrane-bound monomer and at 5 nm from the bilayer center. The results in Figure 8 and Movie S2 reveal a number of features of the dimer assembly process. First, the dimer on the surface assembles rapidly- in a time span below 100 ns- as is shown by the rapid drop in distance between the monomers in Figure 8a. Second, both monomers within the dimer undergo conformational transitions (Figure 8b). Third, the conformational transitions are accompanied by the formation of multiple contacts between the two monomers and the increase in interfacial surface area. The central part of the surface bound monomer, Mon 1, and the C-terminal segment of the free monomer, Mon 2, play the major role in this transition (Figure 8c and Figure S5a). Fourth, similar to the results for the monomer and preformed dimer (Figure 6 and Figure 7), transient interactions of the dimer with the bilayer, primarily via Mon 1, are accompanied by conformational changes (Figure 8c and Figure S5b).

Compared to the preformed dimer, the on-surface dimer shows similar stability; however, it has a smaller interfacial area, is overall less structured with the β-structure content being <20%, and shows a dramatically different interaction pattern. In particular, the interaction pattern shows that while some of the same regions are responsible for stability in the two dimer species, the on-surface dimer is more prone to interactions between the central region and the C-terminal, shown in Figure S5a. The probability of surface interactions is also skewed in favor of the initial surface-bound monomer, whereas the preformed dimer showed no significant difference in preference between the two monomers.

#### 2.2.4. Interactions with POPS Bilayer

Simulations were also performed with POPS bilayers to identify the role of bilayer composition on the Aβ42 interaction and aggregation. The Aβ42 monomer rapidly interacts with POPS and undergoes structural transition into a conformation with β-structure, depicted in Figure S6a. The monomer interacts transiently with the bilayer; however, adsorption (around 2.4 μs for ~600 ns duration) to the bilayer is also observed, Figure S6b. Bilayer interactions are primarily through the N-terminal and residues 23–33, shown in Figure S6c.

Interaction between a POPS-bound (Mon1) and a free (Mon2) Aβ42 monomer happens after ~520 ns and results in a stable dimer, seen in Figure S7a. The formation of the dimer causes a dramatic increase in the β-structure content and a decrease in the helical content, depicted in Figure S7b. This change is further enhanced around ~2.6 μs and results in a final β- structure content of ~20%. The dimer interacts with the POPS bilayer through the N- and central regions of the monomers, shown in Figure S8a. The dimer is stabilized primarily by N-C and C-C terminal interactions, depicted in Figure S8b.

An already formed dimer interacts with the POPS bilayer and undergoes structural transitions, with both monomers within the dimer experiencing an increase in β-structure, shown in Figure S9a. This leads to a dimer interface with the inter-peptide β-sheet. Interactions between the monomers within the dimer are primarily focused around the residues of N–C termini and the central-central region, depicted in Figure S9b. Surface interactions are transient and primarily involve residues in the N-terminal and central regions of the proteins, seen in Figure S9c.

## 3. Discussion

The interaction of Aβ proteins with membranes is widely recognized as a problem of great importance. One of the major foci in previous studies was the ability of Aβ aggregates to make pores within membranes. The formation of these aberrant membrane channels is considered to be among the neurotoxic effects of Aβ aggregates [28,29]. Another interest in the interaction of Aβ proteins with membranes was the elevated rate of assembly of aggregates- primarily fibrils [30,31]; however, the role of membranes in the assembly of oligomers remains unexplored. We have recently discovered a novel property of surfaces, including phospholipid bilayers, in which surface plays the role of a catalyst enabling the assembly of oligomers to occur at nanomolar concentrations [19,20,21,22]. This finding is of great importance, as concentrations of Aβ proteins in vivo are in the low nanomolar concentration range. The model developed in [21] and validated by AFM experiments suggests that aggregation propensity of the on-surface aggregation is defined by the affinity of the protein to the surface and can exceed the bulk aggregation rate by several orders of magnitude. Recent combined experimental and computational studies of the on-membrane aggregation of α-syn revealed a number of properties of the α-syn oligomers assembly on membrane bilayers, including the critical role of the bilayer composition [20,22].

In the present study, we have used time-lapse AFM imaging along with all-atom MD simulations to characterize the self-assembly process of Aβ42 on POPC and POPS bilayers. Experimental studies demonstrate that Aβ42 aggregation occurs at concentrations as low as 10 nM, which is in the range of Aβ42 in vivo concentration and more than three orders of magnitude less than the Aβ42 concentration required for the spontaneous self-assembly of aggregates in test tubes. All previously obtained experimental data were limited to very high concentrations of Aβ42, and this issue was the major concern of the ACH [13,32], which is currently the main model for the development of AD. The on-surface aggregation pathway eliminates this fundamental problem with the ACH.

We then characterized the effect of chemical milieu on the self-assembly process and how the sizes and number of aggregates assembled on the surface increased over time. At ambient conditions and neutral pH values, ionic conditions contribute to the aggregation process, in such a way that aggregation is faster at salt concentrations close to physiological, 150 mM NaCl (Figure 4a,b). A comparison of Aβ42 oligomers assembly on the POPC and POPS bilayers revealed an elevated propensity of POPS compared to POPC, but the strongest effect was observed for the bilayer composed of the two phospholipids in an equimolar ratio (Figure 3). These data lead to the observation that the propensity of Aβ42 to the membrane bilayer surface plays a major role during the self-assembly.

The on-surface aggregation process is dynamic. Oligomers assembled on the surface can dissociate into the solution; moreover, their concentration increases over time (Figure 5 and Figure S3), suggesting that on-surface aggregation is the mechanism by which Aβ42 oligomeric species in solution are produced. Aβ oligomers are widely regarded as the most neurotoxic species, initiating neuron damage and eventually leading to Alzheimer’s disease [32,33] via various pathways, as reviewed in [34]. The disease initiation and development require the accumulation of Aβ oligomers in the brain, and we posit that on-membrane self-assembly is the molecular mechanism for such an accumulation process.

Computational modeling provides important details of the molecular mechanism of the Aβ42 oligomerization catalyzed by the membrane bilayer. According to the MD simulation, interaction with the membrane surface rapidly changes the conformation of the Aβ42 monomer by the formation of extended β-structure motifs (Figure 6). Which are considered major building blocks within Aβ proteins facilitating their assembly into amyloid aggregates (e.g., as reviewed in [35]) and this conformation is termed as a misfolded state. This model was directly confirmed by our simulations of the assembly of Aβ42 dimer through the interaction of unstructured Aβ42 monomer with the surface-bound monomer. The data in Figure 8 demonstrate that misfolded Aβ42 monomer rapidly dimerizes when another monomer appears in proximity to the misfolded one. In turn, as is illustrated in Figure 7, the pre-formed dimer undergoes conformation changes after interaction with the bilayer. Importantly, neither monomers nor dimers stay firmly bound to the bilayer surface; rather, they are dynamic and mobile, able to dissociate, associate, and tumble over the surface, exposing different segments of the molecules to the surface. Evidently, this dynamic behavior further facilitates the misfolding process of either the monomer or dimer.

As we mentioned above, damage to the membranes caused by Aβ42 (including the formation of pores) is considered a major mechanism of the neurotoxicity of Aβ42 oligomers [36]. However, our simulations did not reveal changes in the bilayer surface, neither by monomers nor dimers of Aβ42. We also did not observe any damage to the bilayers during incubation with Aβ42 over a long time period. Moreover, no changes to the surface morphology were identified after the oligomers dissociated from the surface. These findings are in line with results presented in [36], in which no pores were observed for Aβ42 monomers. Nor did they observe pores when 50 nM Aβ42 oligomers were used; however, they were able to detect pores when Aβ42 oligomers at concentrations above 0.5 mM were used.

**Figure 9:**
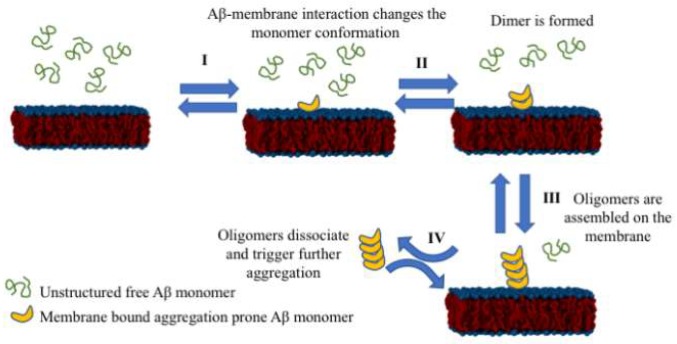
Schematic for the surface-mediated amyloid aggregation model. Amyloid monomers misfold upon interaction with the membrane. In turn, the misfolded dimer assembles by docking to the misfolded monomer. The process continues resulting in the formation of the oligomer, which can dissociate from the membrane to start oligomer-mediated neurotoxic events, defining the disease state at the organism level.

Based on these data, we propose the following model for the assembly of oligomers from monomers, shown in Figure 9, in which cellular membranes play a key role in initiating the aggregation process. In this model, the amyloid monomer changes its conformation upon interaction with the membrane. Therefore, another monomer can interact after the induced conformational change and assemble a dimer. The aggregate grows upon further on-surface docking of additional monomers. Collectively, we termed this process aggregation, catalyzed by interaction with the surfaces. Aggregates can dissociate from the surface and initiate disease-related effects via different pathways [7,36,37,38,39,40,41,42], including interference with recently identified mitochondrial protein biogenesis [43,44] and neuronal hyperactivation at the prodromal state of the disease [4]. Notably, our model is focused on the very early stages of amyloid aggregation, which represent the early onset of disease.

The proposed model is a significant departure from the current ACH model and has three key features. First, it does not require an increase in protein synthesis to the level required for the spontaneous assembly of in vitro aggregates (orders of magnitude higher compared to the in vivo concentrations of amyloids). It explains why attempts at lowering the protein level did not succeed [45,46]- aggregation on membrane surfaces can occur in the physiological range of concentrations, so aggregates can assemble even at very low amyloid concentrations. Second, Aβ, α-syn, and other amyloidogenic proteins are actively involved in important physiological processes such as signal transduction in neuron synapses [42,47,48]. Reducing their levels can impair these important processes [13,16,32,48,49,50]. Third, the composition of the membrane contributes to its interaction with amyloid proteins [51]. Thus, the membrane composition can be the key factor that triggers the aggregation process, and therefore it defines the disease state [52,53,54]. We suggest that preventative and treatment strategies should focus on controlling the membrane composition and interaction of amyloids with membranes.

## 4. Materials and Methods

### 4.1. Materials

Amyloid β (1-42) (Aβ42) was purchased from AnaSpec, Fremont, CA, USA. 1-Palmitoyl-2-oleoyl-sn-glycero-3-phosphocholine (POPC) and 1-palmitoyl-2-oleoyl-sn-glycero-3-phospho-l-serine (POPS) were obtained from Avanti Polar Lipids, Inc, Alabama, USA; Chloroform (Sigma-Aldrich Inc., St. Louis, MO, USA); sonicator (Branson 1210, Branson Ultrasonics, Danbury, CT, USA). Mainly two buffer solutions were used: a 10 mM sodium phosphate buffer with a pH of 7.4 (for without salt condition), and a 10 mM sodium phosphate, 150 mM NaCl, with a pH of 7.4 (for with salt condition). Deionized water (18.2 MΩ, filter pore size: 0.22 μm; APS Water Services Corp., Van Nuys, CA, USA) was used in all the experiments wherever required. Glass vial and glass pipettes (Fisher Scientific, Waltham, MA, USA) were used to handle the lipid solution.

### 4.2. Preparation of Aβ42 Protein Solution

Protein solution was prepared as described previously [19]. Briefly, a measured amount of Aβ42 was dissolved and sonicated for 5 min in 100 μL of 1,1,1,3,3,3-hexafluoroisopropanol (HFIP, Sigma-Aldrich Inc.) to remove any preformed oligomers. The tubes were then put into vacufuge until the solvent is completely evaporated. The stock solution was prepared in DMSO (Sigma-Aldrich Inc.) and kept at −20 °C. Then, 10 nM solution was prepared from the stock in 10 mM sodium phosphate, pH 7.4 just before the experiment.

### 4.3. Preparation of Supported Lipid Bilayers (SLBs)

A similar methodology was implemented to that described previously [20]. Briefly, a 25 mg/mL stock solution of POPC and POPS was prepared in chloroform. Glass vial and glass pipettes were used to handle the lipid solution. Stock solution was stored in −20 °C. An aliquot of 20 μL was taken in another glass vial and dried with a flow of Ar and kept overnight in a vacuum chamber to remove any trace of chloroform. Then, the dried lipid was resuspended in 1 mL 10 mM sodium phosphate, pH 7.4 buffer solution to prepare a 0.5 mg/mL solution which was used for SLB preparation. The solution was gently vortexed for 1 min and then sonicated until the solution became clear. This solution was deposited onto a freshly cleaved mica surface, which was then attached to a glass slide and incubated for 1 h at 60 °C. After the incubation, the slide was cooled to room temperature, the excess of the lipid solution was removed, and the substrate was rinsed with the buffer gently. The prepared SLB was never allowed to dry by keeping ~300 μL buffer on top of it.

### 4.4. AFM Imaging and Data Analysis

Time-lapse AFM imaging was performed in MFP-3D (Asylum Research, Santa Barbara, CA) instrument in tapping mode. MSNL AFM probe (cantilever ‘E’) was used for all the experiments. The nominal resonance frequency and the spring constant of the probe were 7–9 kHz and 0.1 N/m, respectively. The typical scan speed was kept at 1–2 Hz. For time-lapse imaging, images of the same area of the surface were acquired at different time-points. Typically, continuous scanning was avoided. Between each time-point, the cantilever was electronically retracted by the software and engaged again to record the image for the next time-point.

All the AFM images shown were subjected to minimum processing. Only flattening (1st order polynomial) was performed by Femtoscan software (Advanced Technologies Center, Moscow, Russia). The volume of the aggregates was measured by the ‘Grain analysis’ tool of the software and then the distribution of the volume was obtained by plotting them in histogram and fitting the histogram with Gaussian distribution using Origin Pro software (OriginLab, Northampton, MA, USA). Standard deviation was obtained as a half-width of the distribution.

### 4.5. Computational Methods

#### 4.5.1. Molecular Dynamics Simulation of Bilayers

To generate the initial bilayers of POPC and POPS, we employed CHARMM-GUI [55] to produce bilayers consisting of 512 lipid molecules with 40 TIP3P waters [56] per lipid. The lipid systems were neutralized and kept at a 150 mM ionic concentration using Na and Cl counterions and converted to AMBER format using the lipid17 force field (an extension and refinement of lipid14 [57]). The systems then underwent steepest-descent energy minimization, following which 150 ns isothermal-isobaric ensemble (NPT) MD simulations were performed using a 2 fs integration time step. The simulations employed periodic boundary conditions with a semi-isotropic pressure coupling at 1 bar, a constant temperature of 300 K, non-bonded interactions truncated at 10 Å, and electrostatic interactions treated using particle-mesh Ewald [58]. Simulations were performed using the Amber16 package [59].

#### 4.5.2. Molecular Dynamics Simulation of Aβ42 Interactions with Bilayers

To investigate the interaction of Aβ42 monomer with the bilayers, we extracted the POPC and POPS bilayers from the final frame of the pure bilayer simulations, added Aβ42 molecules (monomer or dimer conformations adopted from [27]) at 5 nm center-of-mass (CoM) from the bilayer center, solvated in TIP3P water (in an orthorhombic box [a = b ≠ c] with side:height ratio 0.75), neutralized with NaCl counter ions, and maintained a final NaCl concentration of 150 mM. Proteins were described using the Amber ff99SB-ILDN force field [60]. Each system then underwent H-mass repartitioning to increase the H mass to 3.024 Da allowing for 4 fs time steps [61], following which the systems were simulated as an NPT ensemble for 5 ns (using the same parameters as pure bilayer simulations) before being submitted to the special purpose supercomputer Anton2 for long production runs. Simulations on Anton2 employed the multigrator algorithm and treated electrostatics using the Gaussian split Ewald method.

#### 4.5.3. Interaction between Membrane-Bound and free Aβ42 Monomer

To investigate the interaction between membrane-bound and free Aβ42 monomers, we used the last membrane-bound conformation of the previous simulation systems and added a monomer to the simulation systems following the same procedure as used initially to add Aβ42 molecules to the bilayer systems. Newly added molecules were placed at 4 nm CoM with respect to the membrane-bound molecule and at 5 nm distance to the membrane core. Simulation parameters and steps were the same as the initial Aβ42-bilayer simulations.

#### 4.5.4. Analysis of MD Trajectories

Gromacs suite of programs (v2016) [62] was used to analyze the obtained simulation trajectories.

## Figures and Tables

**Table 1:** Description and nomenclature of simulated bilayer systems.

Name	Bilayer	Aβ42 Species	Duration [μs]
PC	POPC′	-	0.150
PS	POPS″	-	0.150
PCm	POPC	Monomer	5
PSm	POPS	Monomer	5
PCmm	POPC	Bound monomer + monomer	5
PSmm	POPS	Bound monomer + monomer	5
PCd	POPC	Dimer	5
PSd	POPS	Dimer	5

′1-Palmitoyl-2-oleoyl-sn-glycero-3-phosphocholine, ″1-palmitoyl-2-oleoyl-sn-glycero-3-phospho-l-serine.

## Supplementary Materials

Supplementary materials can be found at https://www.mdpi.com/1422-0067/21/3/1129/s1.

## Abbreviations

Aβ	Amyloid β
ACH	Amyloid cascade hypothesis
AFM	Atomic force microscopy
CSF	Cerebrospinal fluid
CoM	Center of mass
MD	Moleuclar dynamics
POPC	1-palmitoyl-2-oleoyl-glycero-3-phosphocholine
POPS	1-palmitoyl-2-oleoyl-sn-glycero-3-phospho-l-serine
SLB	Supported lipid bilayer
