# Supplementary material for: Interaction of Aβ42 with Membranes Triggers the Self-Assembly into Oligomers
Source: Int J Mol Sci. 2020 Feb 8;21(3):1129. doi: 10.3390/ijms21031129 (PMC7036922; doi:10.3390/ijms21031129)
Supplement: Supplementary file 1 [file ijms-21-01129-s001.zip › AB42-membranes_Supplement_V3_cln.pdf]

Supplementary Materials for

# Interaction of Aβ42 with membranes triggers the self-assembly into oligomers

Siddhartha Banerjee[1,†], Mohtadin Hashemi[1,†], Karen Zagorski[1] and Yuri L. Lyubchenko[1,*]

*Correspondence to: ylyubchenko@unmc.edu †These authors contributed equally

**This PDF file includes:**

Captions for Supplementary Movies 1 and 2

Figures S1 to S9

**Other Supplementary Materials for this manuscript include the following:**

Supplementary Movies 1 and 2

**Movie S1. Interaction of Aβ42 monomer with the POPC bilayer surface.** The bilayer surface facilitates the transition of the protein structure from a conformation with small β-structure content to a conformation with an extended β-sheet consisting of two strands, which remains stable for the rest of the simulation. Protein side-chains and POPC head groups are shown for interacting residues, protein is depicted as cartoon following VMD coloring scheme (yellow β-strands and purple α-helices) , N-terminal Cα is represented as a large blue sphere, and POPC P atoms are depicted as gold spheres.

**Movie S2. Interaction of membrane-bound Aβ42 monomer with a free monomer on POPC.** Transient interaction between the initially free monomer and the membrane surface are observed, but the monomer remains free, which is followed by diffusion above the bilayer toward the bound Aβ42 monomer. After a short period, the two monomers interact and form a dimer. The conformation of both monomers changes dramatically during the on-surface dimerization. Protein side-chains and POPC head groups are shown for interacting residues, protein is depicted as cartoon following VMD coloring scheme, N- and C-terminal Cα are represented as large and small spheres in blue and red for Mon1 and Mon2, respectively, POPC P atoms are depicted as gold spheres.

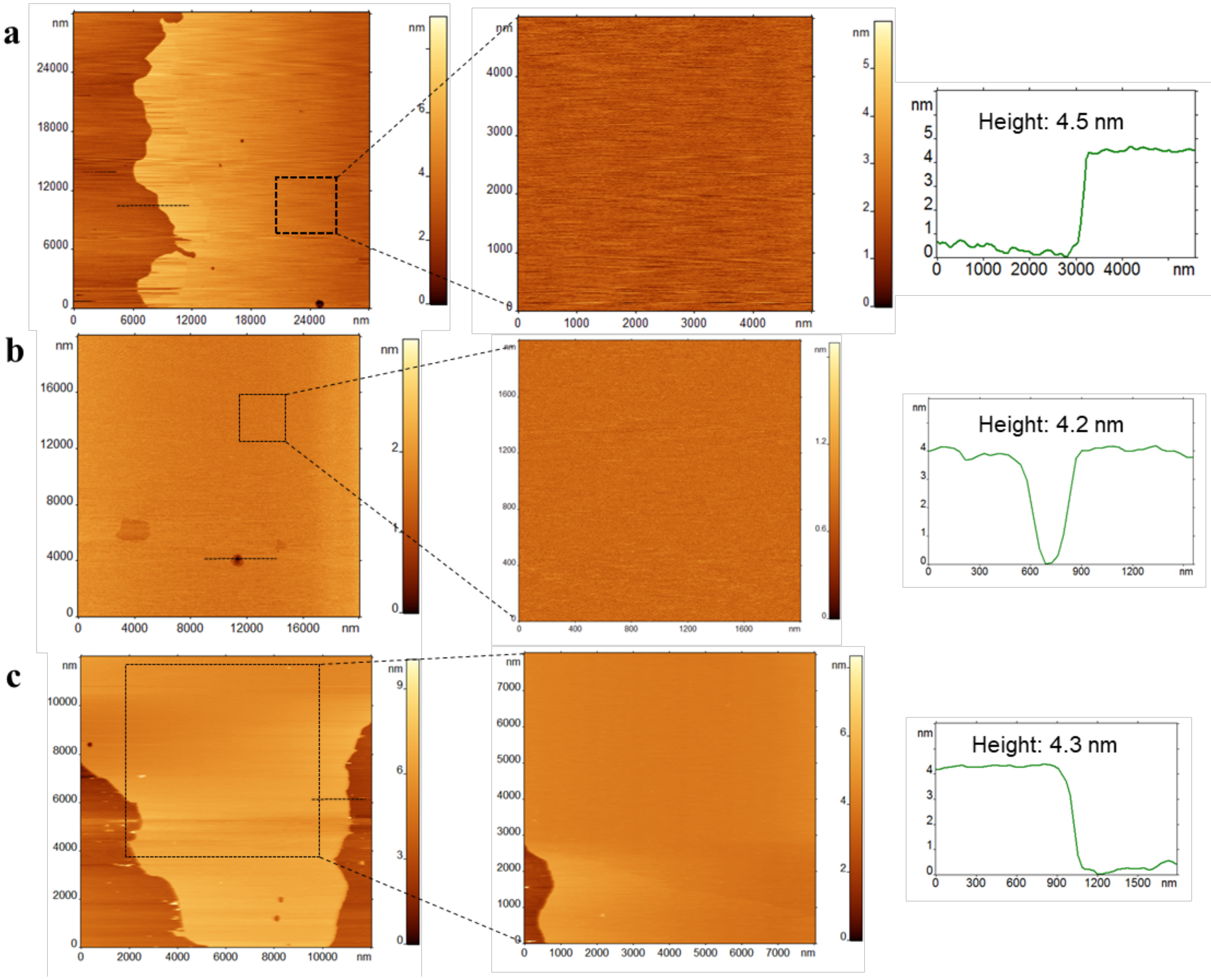


**Figure S1. Representative AFM images of supported lipid bilayers used in this study.** AFM topographic images showing large, smooth SLB surfaces of (**a**) POPC, (**b**) POPS and (**c**) POPC-POPS mixture (1:1 mol). The first column shows the large scans and scans for zoomed area have been shown in the second column. The SLB surfaces are smooth and devoid of any unruptured or trapped vesicles. Third column shows the cross-section profiles, which indicate the height of the prepared bilayer. The height of the bilayer remains 4.2-4.5 nm.

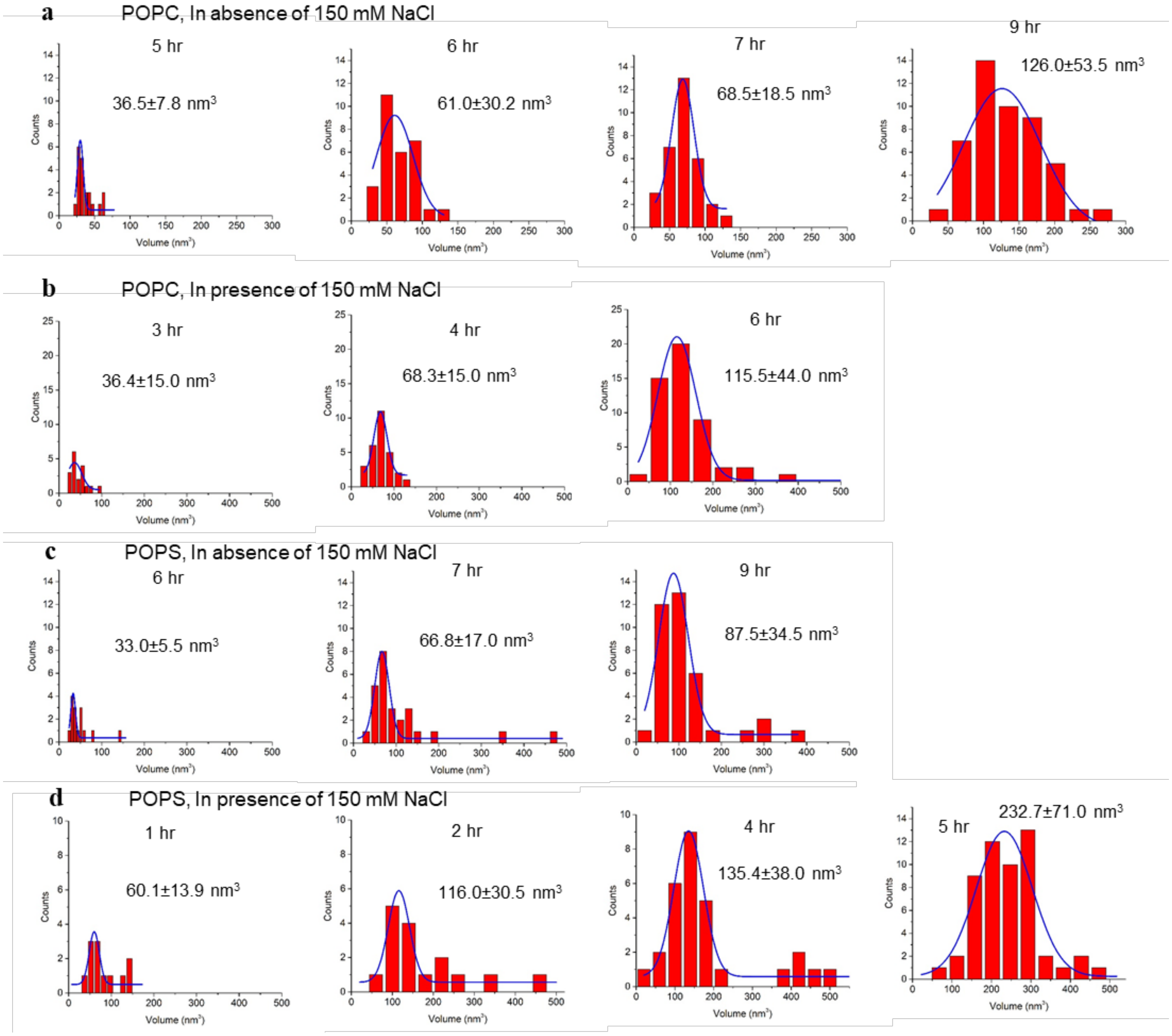


**Figure S2. Quantitative analysis of 10 nM Aβ42 aggregation on POPC and POPS SLBs in absence or presence of 150 mM NaCl.** (**a**) and (**b**) show the volume distribution of the aggregates formed at different time points on the POPC SLBs in the absence and presence of 150 mM NaCl, respectively. (**c**) and (**d**) show the aggregate volumes on POPS SLBs in the absence and presence of 150 mM NaCl, respectively.

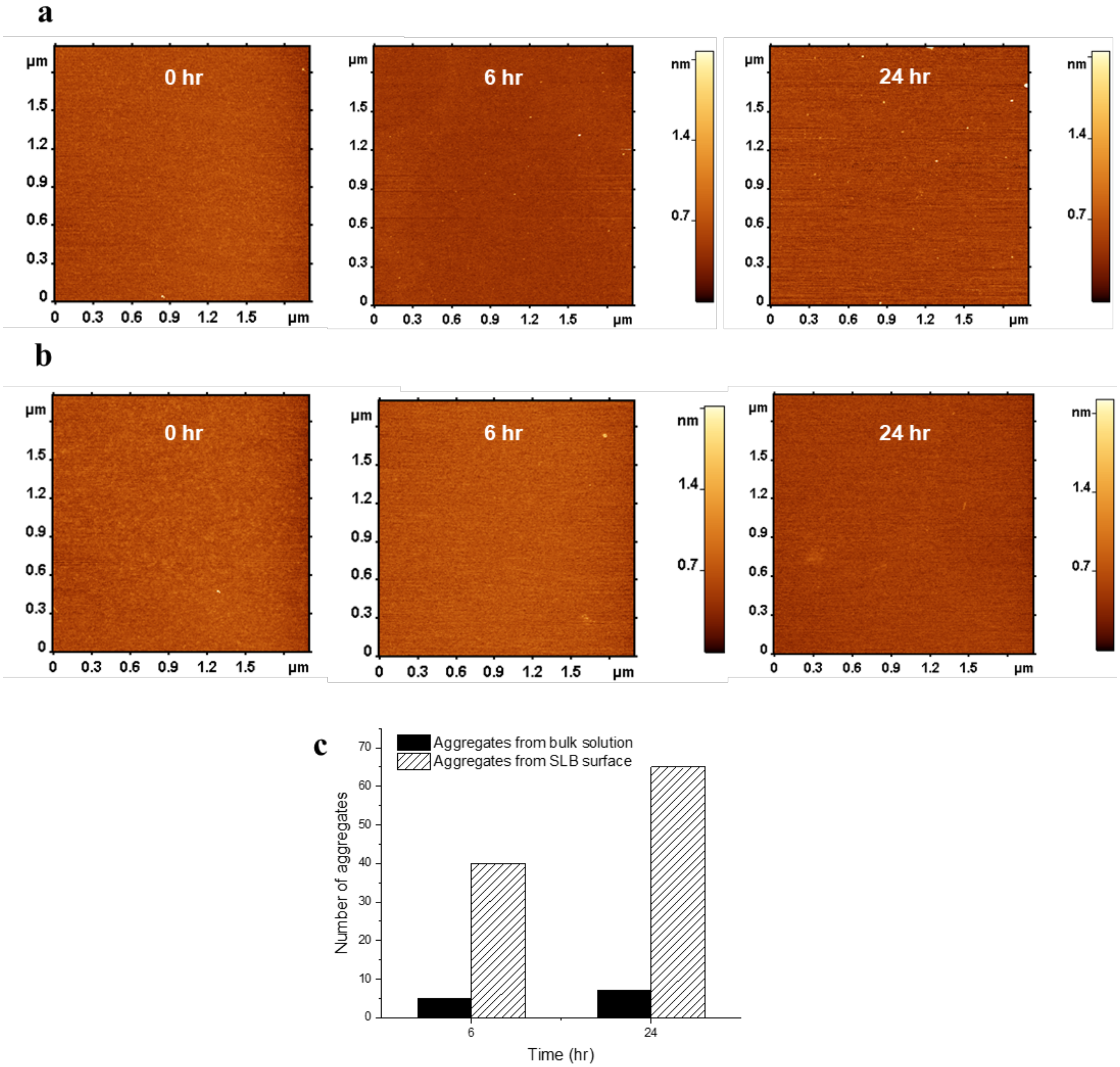


**Figure S3. Bulk experiment showing the dissociation of the aggregates from the SLB surface.** AFM images of Aβ42 aggregates present in bulk solution at different time intervals (**a**) in presence of POPS SLB surface and (**b**) in absence of SLB surface. AFM images clearly show the presence of higher number of aggregates at 24 hr panel in the presence of SLB surface compared to its absence. (**c**) The bar diagram shows the comparison in the number of aggregates present in two situations. The numbers are computed from two 2 µm × 2 µm images for each time point.

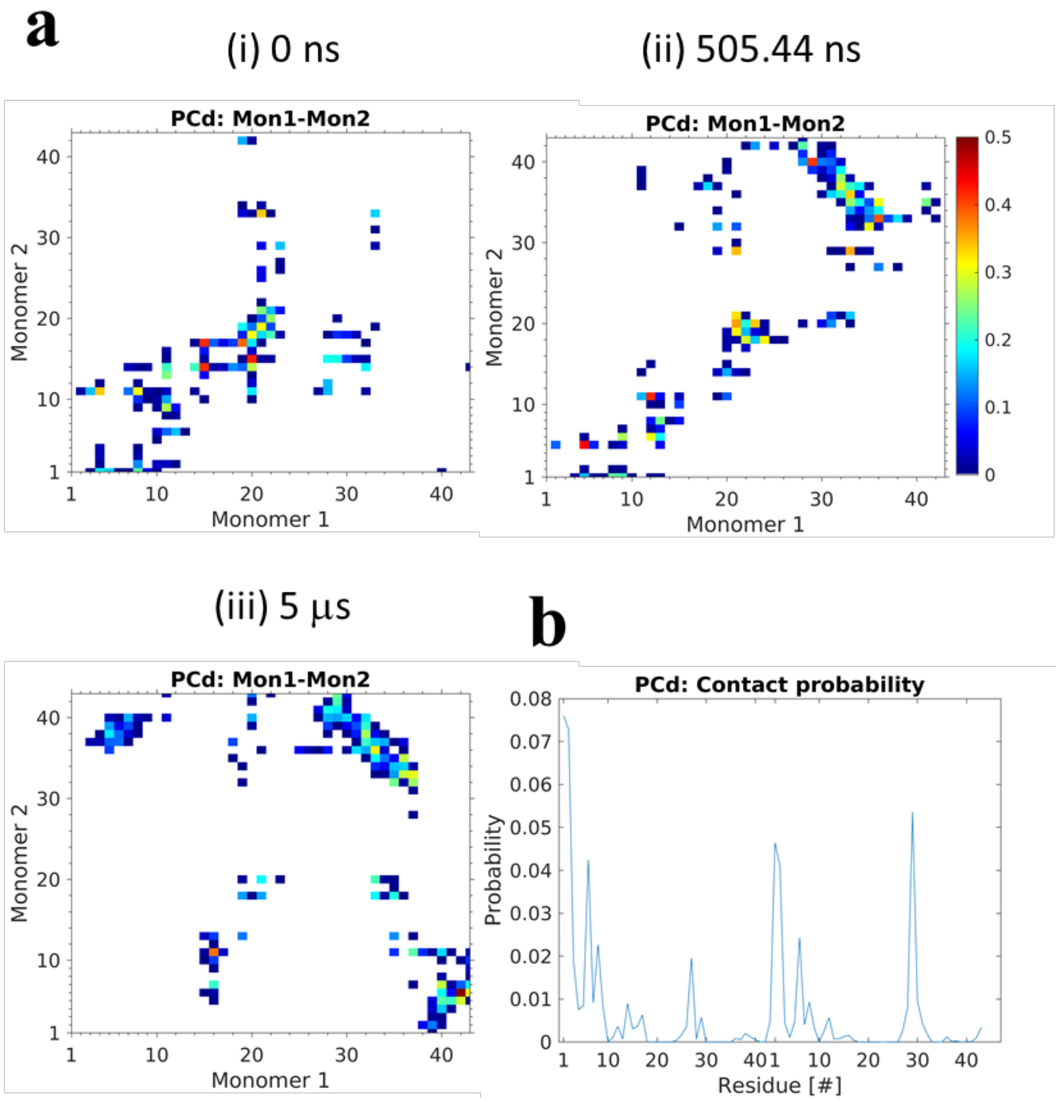


**Figure S4. Analysis of the interaction of an Aβ42 dimer with POPC bilayer (PCd system).** (**a**) Normalized contact map based on the contacts of individual residues interacting with the opposite monomer; color represent number of contacts between the residue pairs. (**b**) Interaction probability between protein residues and P atoms of the lipid headgroups.

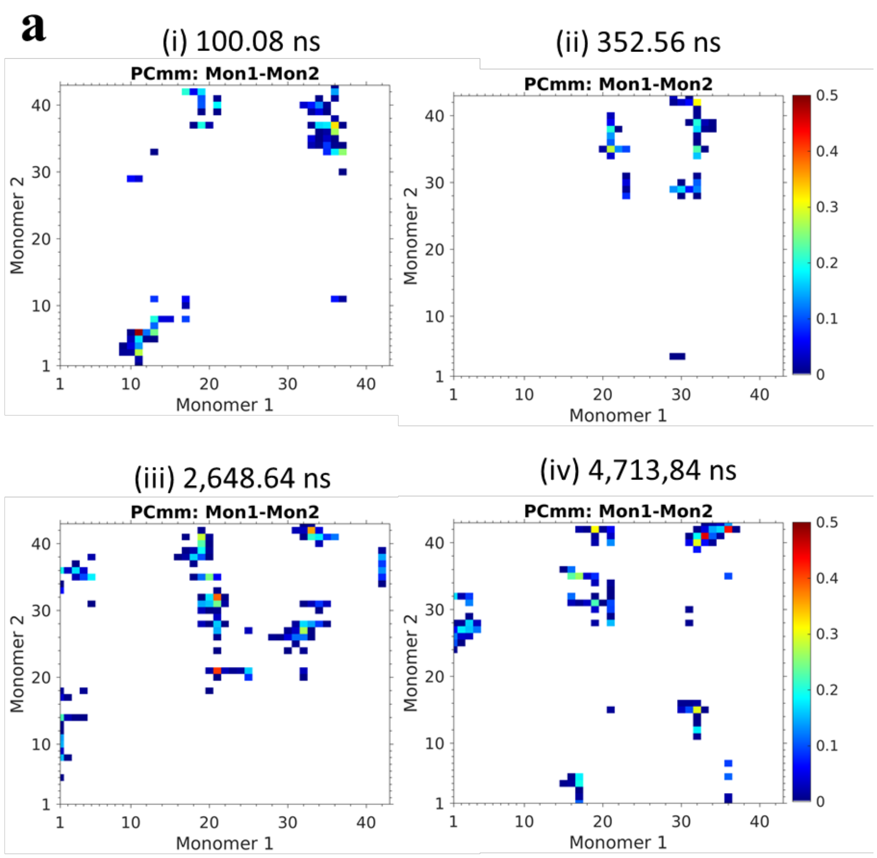


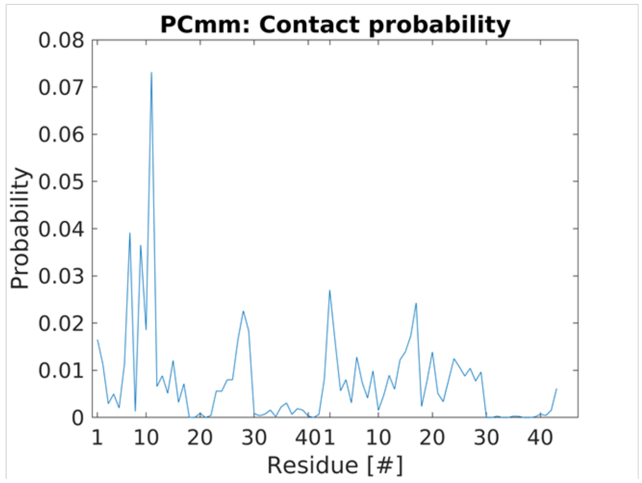


**Figure S5. Analysis of contacts during the interaction of membrane-bound and free Aβ42 monomers with POPC bilayer (PCmm system).** (**a**) Normalized contact map based on the contacts of individual residues interacting with the opposite monomer; color represent total number of contacts between the residue pairs. (**b**) Interaction probability between protein residues and P atoms of the lipid headgroups.

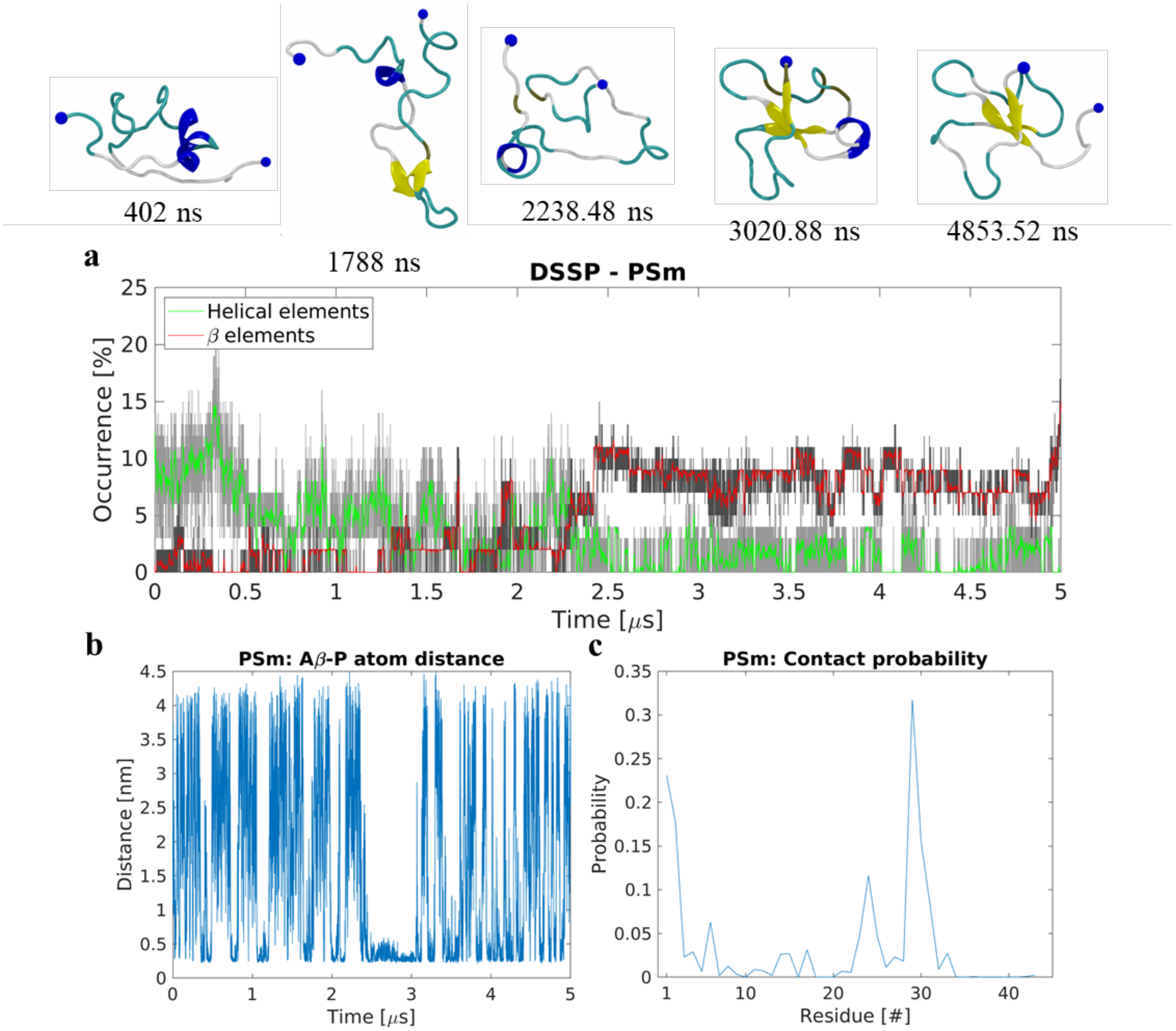


**Figure S6. Interaction of Aβ42 monomer with POPS bilayer (PSm system).** (**a**) Evolution of secondary structure β- (sheet and bridge), red, and helical (α-, π-, and 3/10-helices), green, elements as determined by DSSP. The graphs are moving averages using a 1 ns window; raw data is presented as dark and light grey graphs, respectively. Snapshots show the adoption of a transient N-terminal α-helix that is later converted into a small helical segment before the monomer finally adopts a small three-strand β-sheet. Colors follow VMD coloring scheme (yellow β-strands and purple α-helices), N- and C-terminal Cα are represented as a large and a small spheres, respectively. (**b**) Center of mass distance between the monomer and the P atoms of the lipid headgroups. (**c**) Normalized contact map based on the contacts of individual residues interacting with the opposite monomer; color represent total number of contacts between the residue pairs.

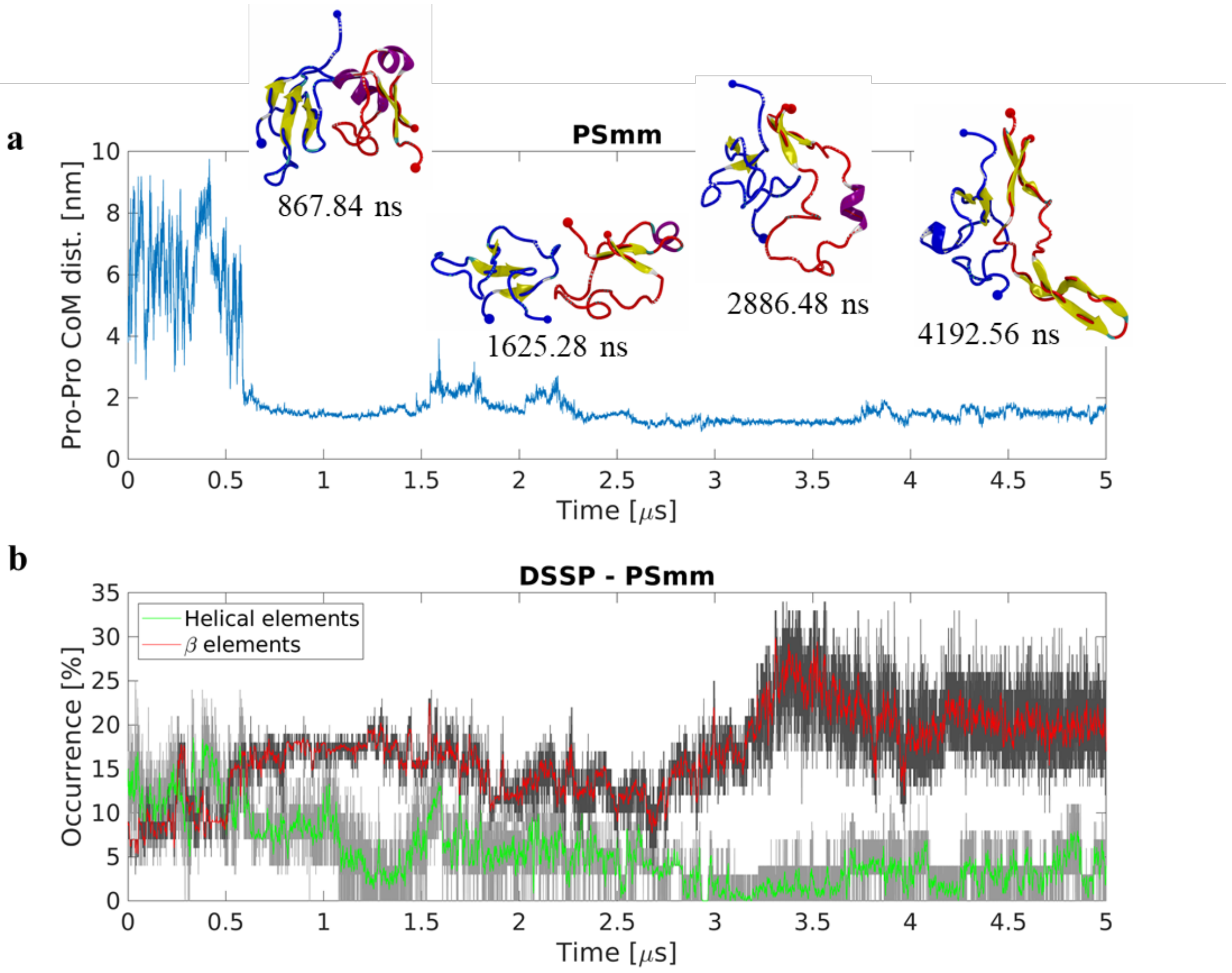


**Figure S7. Interaction of bilayer-bound Aβ42 monomer with free monomer on POPS bilayer (PSmm system).** (**a**) Center of mass distance between monomers in the PSmm system showing the distance between the surface-bound monomer, Mon1, and the free monomer, Mon2. A stable dimer is formed after ~520 ns and remains in the same configuration for the rest of the simulation. Snapshots showing the dynamics of the interaction process and the change in the arrangement and secondary structure of the monomers within the dimer. Colors follow VMD coloring scheme (yellow β-strands and purple α-helices), N- and C-terminal Cα are represented as a large and a small sphere respectively; Mon1 and Mon2 backbone and sphere colors are blue and red, respectively. (**b**) Evolution of secondary structure β- (sheet and bridge), red, and helical (α-, π-, and 3/10-helices), green, elements as determined by DSSP. The formation of the dimer causes a dramatic increase of the β-structure content and a decrease in the helical content. This change is further enhanced around ~2.6 μs and results in a final β-structure content of ~20%. The graphs are moving averages using a 1 ns window; raw data is presented as dark and light grey graphs, respectively.

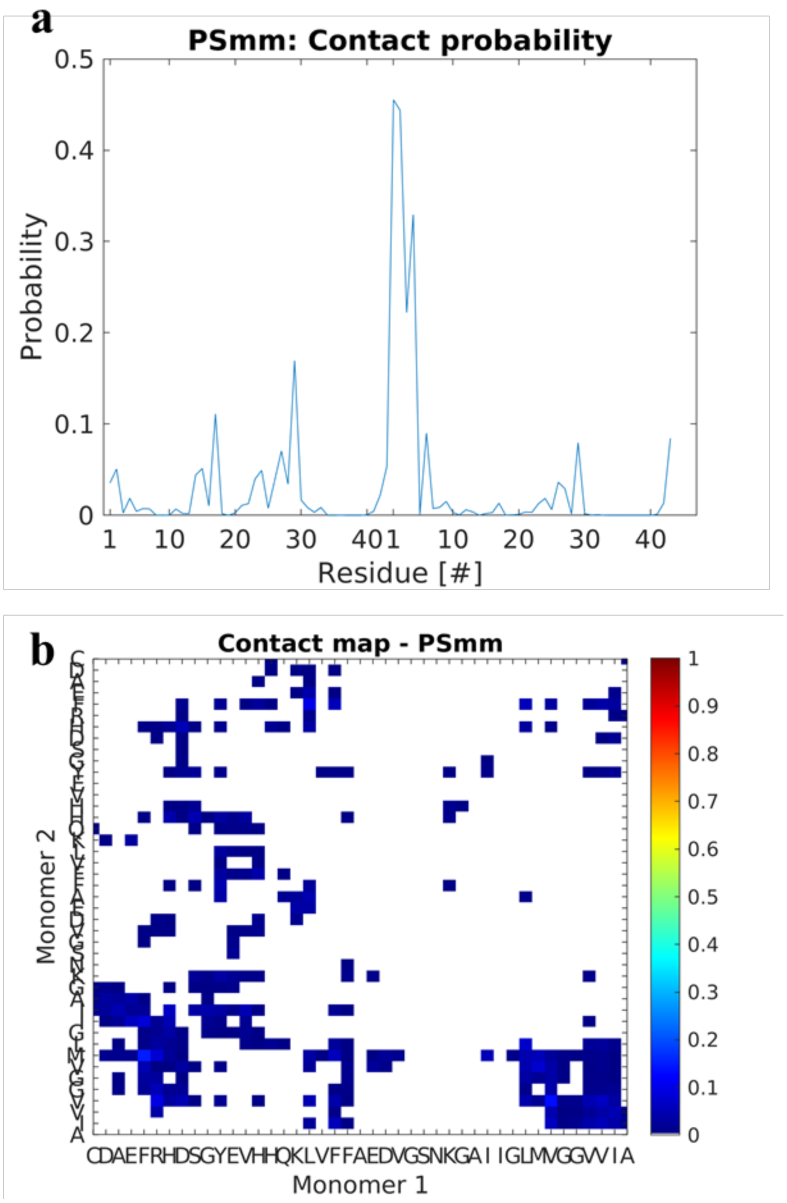


**Figure S8. Analysis of contacts during interaction of bilayer-bound Aβ42 monomer with free monomer on POPS bilayer (PSmm system).** (**a**) Interaction probability between protein residues and P atoms of the lipid headgroups. (**b**) Normalized per-residue contact map showing that the dimer is stabilized through N-C and C-C interactions with a significant contribution from N-central region interactions as well. Colors represent total number of contacts (<6 Å) between the residue pairs.

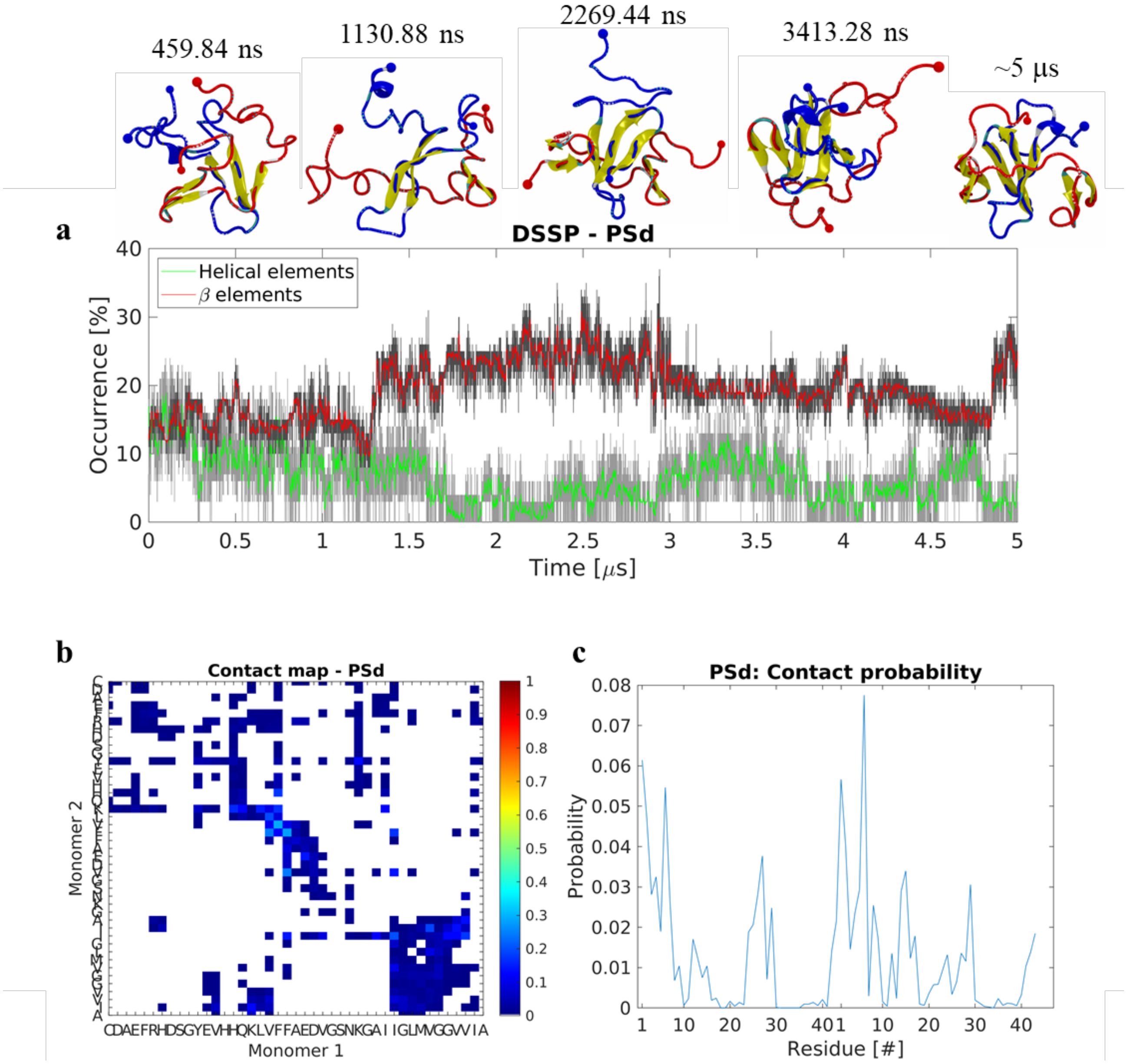


**Figure S9. Interaction of Aβ42 dimer with POPS bilayer (PSd system). (a)** Evolution of secondary structure β- (sheet and bridge), red, and helical (α-, π-, and 3/10-helices), green, elements as determined by DSSP showing that the dimer undergoes a stepwise increase in β-content while helical content fluctuates between 1-10%. The graphs are moving averages using a 1 ns window; raw data is presented as dark and light grey graphs, respectively. Snapshots show the change in monomer arrangement and secondary structure of the Aβ42 molecules as cartoon representation following VMD coloring scheme (yellow β-strands and purple α-helices). N- and C-terminal Cα are represented as a large and a small sphere respectively; blue and red sphere and backbone colors represent Mon1 and Mon2, respectively. (**b**) Normalized per residue contact map, showing the interaction between the monomers within the dimer. (**c**) Interaction probability between protein residues and P atoms of the lipid headgroups.
